# A first report of *Biomphalaria pfeifferi* in the Lower Shire Valley, Southern Malawi, a major intermediate snail host species for intestinal schistosomiasis

**DOI:** 10.1038/s41598-025-88930-4

**Published:** 2025-02-15

**Authors:** Clinton Nkolokosa, Rex Mbewe, James Chirombo, Michelle C. Stanton, Christopher M. Jones, Peter Makaula, Gladys Namacha, Blessings Chiepa, Patrick Ken Kalonde, Chifuniro Baluwa, Kennedy Zembere, Eggrey Aisha Kambewa, Chikumbusko Chiziwa Kaonga, John Archer, Alexandra Juhász, Lucas J. Cunningham, Julie-Anne Akiko Tangena, J. Russell Stothard

**Affiliations:** 1 Malawi-Liverpool-Wellcome Programme, Blantyre, Malawi; 2 Liverpool School of Tropical Medicine, Liverpool, L3 5QA UK; 3 Malawi University of Business and Applied Science, Blantyre, Malawi; 4 University of Stirling, Stirling, FK9 4LA UK

**Keywords:** DNA, Ecological epidemiology, Freshwater ecology, Genotype, Haplotypes, Sequencing

## Abstract

The distribution of certain permissive intermediate snail host species in freshwater is a crucial factor shaping transmission of intestinal schistosomiasis, a neglected tropical disease that causes much human suffering in Africa. To shed new light on southern Malawi, where cases of intestinal schistosomiasis have been found, repeated malacological surveys were conducted in Chikwawa and Nsanje Districts in the Lower Shire Valley, to detect and to characterize populations of *Biomphalaria*, the intermediate host for intestinal schistosomiasis. Sampling took place across a total of 45 freshwater sites, noting water conductivity, pH, temperature, total dissolved salts (TDS) and geographical elevation. The presence or absence of snails was predicted upon physiochemical and environmental conditions in Random Forest modelling. Water conductivity, TDS and geographical elevation were most important in predicting abundance of snails with water temperature and pH of slightly less important roles. This first report of *B. pfeifferi* in the Lower Shire Valley enhances understanding of the environmental factors that strongly associate and allow prediction of its local distribution. This represents a useful step towards developing appropriate intervention strategies to mitigate intestinal schistosomiasis transmission.

**Supplementary Information:**

The online version contains supplementary material available at 10.1038/s41598-025-88930-4.

## Introduction

Several freshwater snail species within the genus *Biomphalaria* (Gastropoda: Planorbidae) serve as obligatory intermediate hosts of *Schistosoma mansoni* (Trematoda: Schistosomatidae), a parasitic blood fluke that causes intestinal schistosomiasis^1^. Among these snail species, *Biomphalaria pfeifferi* is most often regarded with greatest medical interest in sub-Saharan Africa^1^ for it is considered a major intermediate host, and often an invasive species, which facilitates disease transmission in many African countries^2–4^. In East and Central Africa, *B. pfeifferi* is commonly found predominately in riverine environments within Uganda^5^, Kenya^3,6^ and Tanzania^6^, while in Malawi its distribution is not fully understood^7,8^. Recently, this species has successfully colonized the Lake Malawi shoreline^2,9^. However, until our observations reported here, its occurrence in the Lower Shire Valley was suspected but not confirmed. Of note, a decade ago the prevalence of intestinal schistosomiasis in Chikwawa District, southern Malawi, as determined by urine-cathodic circulating antigen dipsticks, was 9.1% in pre-school-aged children and 24.9% in their mothers, with eggs of *S. mansoni* often confirmed in stool. However, concurrent malacological surveys failed to find *Biomphalaria* locally, despite extensive searching^10^. Prior to this, *B. pfeifferi* has only been confirmed in Dowa District, central Malawi^7^ and around Lake Malawi, specifically Mangochi District^2^, and in the past, in Karonga^2^, a much more northerly location and marginal along the lake.

Draining southwards, the Shire River exits Lake Malawi and flows towards Chikwawa and Nsanje Districts. Despite regulation of water flow for hydroelectricity, for example, seasonal flooding with local formation of potential freshwater snail habitats along both sides of the river may occur. To better understand the epidemiology of intestinal schistosomiasis transmission, repeated malacological surveys were conducted from May 2023 onwards to investigate and characterize the putative occurrence of *Biomphalaria* in the Lower Shire Valley: Chikwawa and Nsanje Districts. Determining the spatial presence of *Biomphalaria* sp. in microhabitats is necessary for identifying focal sources of intestinal schistosomiasis transmission and inform more effective mitigating disease control actions^4,5,11^. In Malawi, the National Schistosomiasis Control Programme provides praziquantel by regular mass drug administration (MDA)^12,13^ and to augment this foundational intervention, it is important to monitor and ascertain the geographical distribution of permissive intermediate snail hosts. Furthermore, better snail surveillance provides essential data on freshwater invertebrate biodiversity, and environmental management needs, against a background of wider climate change and anthropogenic impacts^14–17^.

Our aim was to identify habitats occupied by *Biomphalaria* in the Lower Shire Valley, determining the environmental factors influencing the distribution of these snails. We examined five abiotic factors that may affect the spatial extent of suitable habitats for these snails across the study area.

## Materials and methods

### Study area

**Fig. 1:**
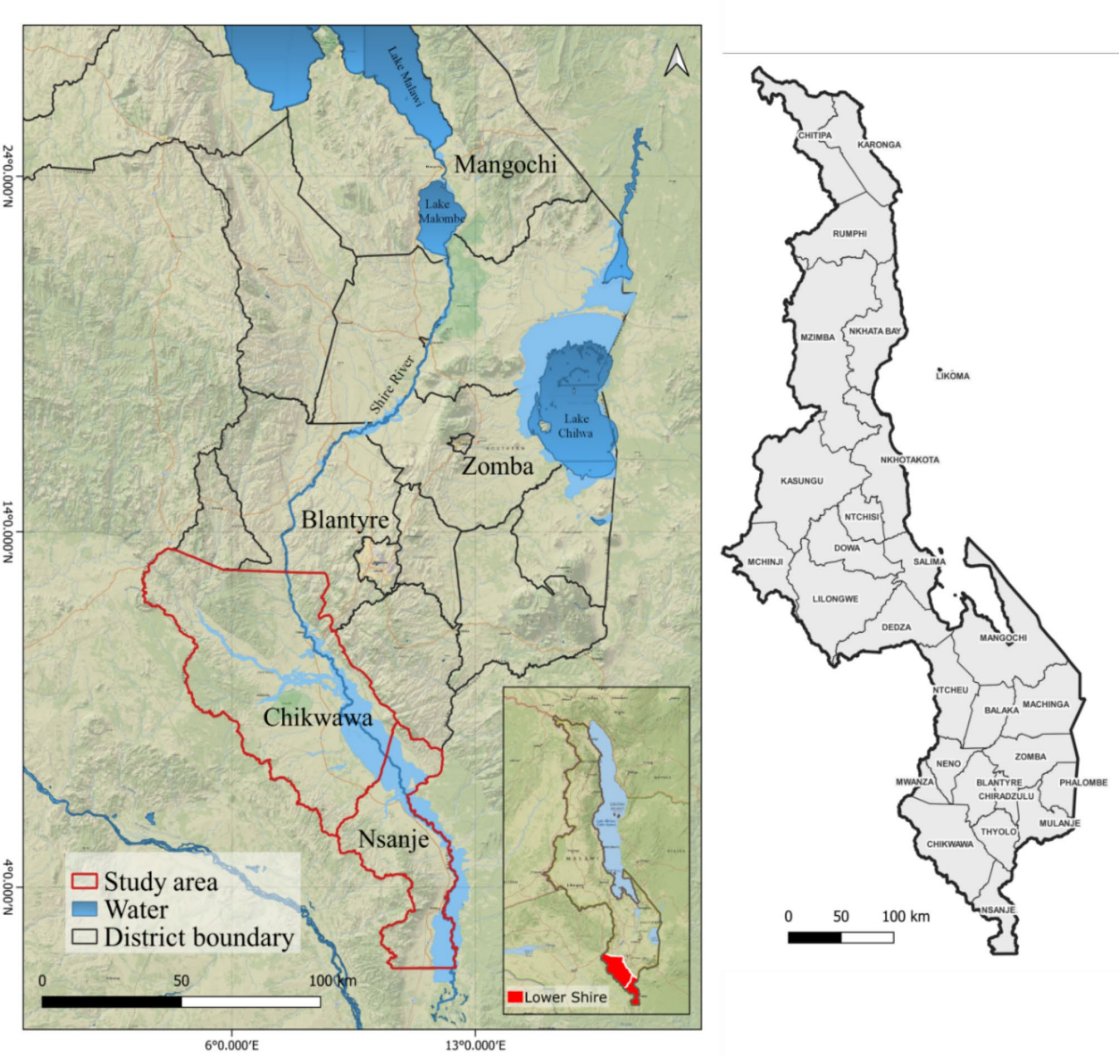
Chikwawa and Nsanje Districts in the Lower Shire of Malawi as presented with QGIS version 3.22.1 mapping software (https://qgis.org/). The inset map shows the location of Lower Shire Valley in the context of Malawi.

The study area covers Chikwawa (coordinates: 34.81265° E, 16.03223° S; land area: 4,878 km^2^) and Nsanje (coordinates: 35.26185° E, 16.92282° S; land area: 1,945 km^2^) districts, located along the lower flat basin of Shire River in southern Malawi, (Fig. 1). Most of Chikwawa and Nsanje have the lowest elevation in the country, ranging from ~ 42 − 1,584 m and ~ 31–965 m above mean sea level, respectively^18,19^. Both districts experience wet and dry tropical climates and are vulnerable to floods and droughts, attributed to climate change^19,20^. In 2018, Chikwawa and Nsanje districts had a population of 564,684 and 299,168 people, respectively^21^. Irrigated agriculture, fishing and subsistence farming in naturally occurring floodplains and riverine wetlands are currently dominant land- and water-use activities^18^. The prevalence of schistosomiasis from urine surveys conducted by the Ministry of Health in Chikwawa in 2008 and Nsanje in 2010 ranged from 14 to 56% and 44–62%, respectively^12^.

### Malacological survey and cercarial shedding analysis

A prospective malacological survey was conducted from the 6th to 15th May 2023 to obtain data on the occurrence of *Biomphalaria*, estimate snail populations (numerical abundance) and measure environmental variables (conductivity, pH, salinity and temperature of water, and elevation) across 45 freshwater habitats in Chikwawa and Nsanje Districts. This study strategically sampled 45 potential freshwater snail habitats (i.e., aquatic microhabitats) in Chikwawa (*n* = 37) and Nsanje (*n* = 18) (Supplementary Table 1). The survey targeted aquatic habitats, such as lakes and marshlands, ponds and pools, rivers and canals, especially where human-water contact was known or observed to occur (including activities such as fishing, gardening, bathing, swimming, washing, etc.). The selection of sampling sites was based on accessibility and prior knowledge that molluscan intermediate hosts of schistosomes occur in lotic and lentic ecosystems and exhibit non-random distribution^22,23^. Thus, using stratified random sampling, we employed a direct method of surveying snails that involved passing a dip net, metal scoop and sieve (fine wire mesh kitchen strainer attached to a metal handle) through water, vegetation and mud to collect freshwater snails for 15 min^24,25^. In addition, the study investigated substrates, including plastic, sticks, stones, living and dead vegetation (e.g. water lily leaves) found in a water body. The morphology of shells was first used to distinguish *Biomphalaria* spp. from other genera of freshwater snails by a trained malacologist^26,27^. Dominant vegetation, water flow and presence of humans, animals and other freshwater snail genera such as *Bulinus*, *Lymnaea*,* Melanoides* and *Lanistes* were noted. However, given the focus on *Biomphalaria* here, it is beyond the scope of this study to examine these other data.

At each collection site, the study obtained geographical coordinates (Universal Transverse Mercator latitude and longitude) and elevation (m) using a hand-held Garmin Global Positioning Satellite (Garmin Montana 700 GPS, US), and water quality data. Water pH, water temperature (°C), conductivity (µS/cm) and total dissolved salts (TDS in ppm) were measured in situ using a combined pH, conductivity, TDS pocket tester: Hanna HI98129 (Hanna Instruments Ltd, UK). The pH, electroconductivity (EC), and TDS (ppm) readings are automatically temperature compensated to prevent temperature-related variations in the measurements. Of note, water samples taken for field measurement were collected immediately prior to collection of snails. This was to avoid disturbing sediments in the immediate sampling area. Additionally, when sampling in wadable waters, care was taken to avoid disturbing the stream bed or riverbed before collection of water in the sample container bottle. Elevation, temperature and water chemistry are generally important factors for the habitation of the *Biomphalaria* snails^5,22,26^. Natural factors (e.g., temperature) and physicochemical parameters of water constrain or expand the distribution of the freshwater snails of the genus *Biomphalaria*^5,22,27^. Against this background, a careful a priori selection of the environmental variables hypothesized to directly influence *Biomphalaria* distribution and abundance in freshwater ecosystems was made. Regarding the choice of the explanatory variables, we used five abiotic factors that are known to affect freshwater snail distribution, namely elevation, pH, temperature, conductivity and TDS^27,28^. To aid habitat reconnaissance, vertical and oblique aerial photographs (20MP camera sensor) and videos (5.1 K video recording) of the semi-aquatic habitat were captured by a small commercial-grade drone, Mavic 3 (DJI Shenzen, China), at an altitude of 40 m with the camera pointed at 90 and 45 degrees downwards and forwards, respectively.

Following malacological collections, all collected *Biomphalaria* were subjected to cercarial shedding analysis in attempt to identify any snails harboring patent infections with *Schistosoma* spp. trematodes according to a standard inspection protocol outlined previously^29^.

### Molecular characterization and molecular xenomonitoring of collected *Biomphalaria*

DNA was isolated from eight randomly selected ethanol preserved *Biomphalaria* specimens using the QIAGEN DNeasy Blood & Tissue Kit [QIAGEN, UK]. These snails originated from Site 17, located within the Nchalo Estate, and were collected from a water site considered to have had the greatest human water contact based upon observations on site at the time of snail collections. DNA extraction was conducted according to manufacturer’s instructions with minor revisions including using double volume of ATL buffer and Proteinase K during tissue lysis^30^.

Following DNA extraction, these eight *Biomphalaria* specimens were characterised to species level using end-point PCR and Sanger sequencing of a 700-bp region of the mitochondrial *cox*1 gene, as detailed previously^9,30^. In addition, a recently developed *S. mansoni*-specific molecular xenomonitoring assay was also used in an attempt to detect patent, but non-shedding, or prepatent *S. mansoni* infections within these eight *Biomphalaria* specimens^29^.

### Environmental data analysis

The study employed a Random Forest (RF) machine learning framework to determine the environmental variables that are directly linked with *Biomphalaria* spatial presence and absence^31^. The RF is resistant to overfitting, does not require cross-validation and provides comparatively excellent accuracy^31–33^. First, the study derived a presence-absence (yes/no) categorical variable from the numerical abundance of the *Biomphalaria* snails at each site. Then a spatially-naive RF classification model was fitted, using the present/absent variable as the response variable and elevation, water pH, temperature, conductivity and TDS as the explanatory variables. In RF, both the predictors and responses are randomly arranged in all possible ways^31,32^. The model was implemented using random Forest package (version 4.7.1.1)^34^ and visualised using reptree package (version 0.6)^35^ in R Statistical Software (version 4.2.2; R Core Team 2022)^36^. Model performance was assessed using accuracy, precision, recall (sensitivity) and F1 metrics calculated from confusion matrices and out-of-bag (OOB) error estimates. The OOB error is an estimate of prediction error on test data^37,38^. The importance of each explanatory variable in predicting *Biomphalaria* absence/presence was measured using mean decrease Gini (impurity) and mean decrease accuracy (permutation importance), MDG and MDA hereinafter, respectively. In an RF, MDG measures how much an input variable reduces the impurity of the tree^39^. The MDA measures the reduction in accuracy of the model when a variable is randomly permuted^40^. As the MDG or MDA values of a variable increase, its importance in the model increases^40^.

In addition, the Wilcoxon test was employed to evaluate the environmental ranges associated with the presence or absence of *Biomphalaria*, based on the statistical significance of the differences represented in the boxplots. This non-parametric approach was chosen due to its ability to accommodate the occurrence data, as it does not presume a normal distribution. The interpretability of the data is improved by incorporating the results of this analysis, which include p-values, into the boxplots.

## Results

### Current occurrence and abundance of *Biomphalaria* determined in Lower Shire

**Fig. 2:**
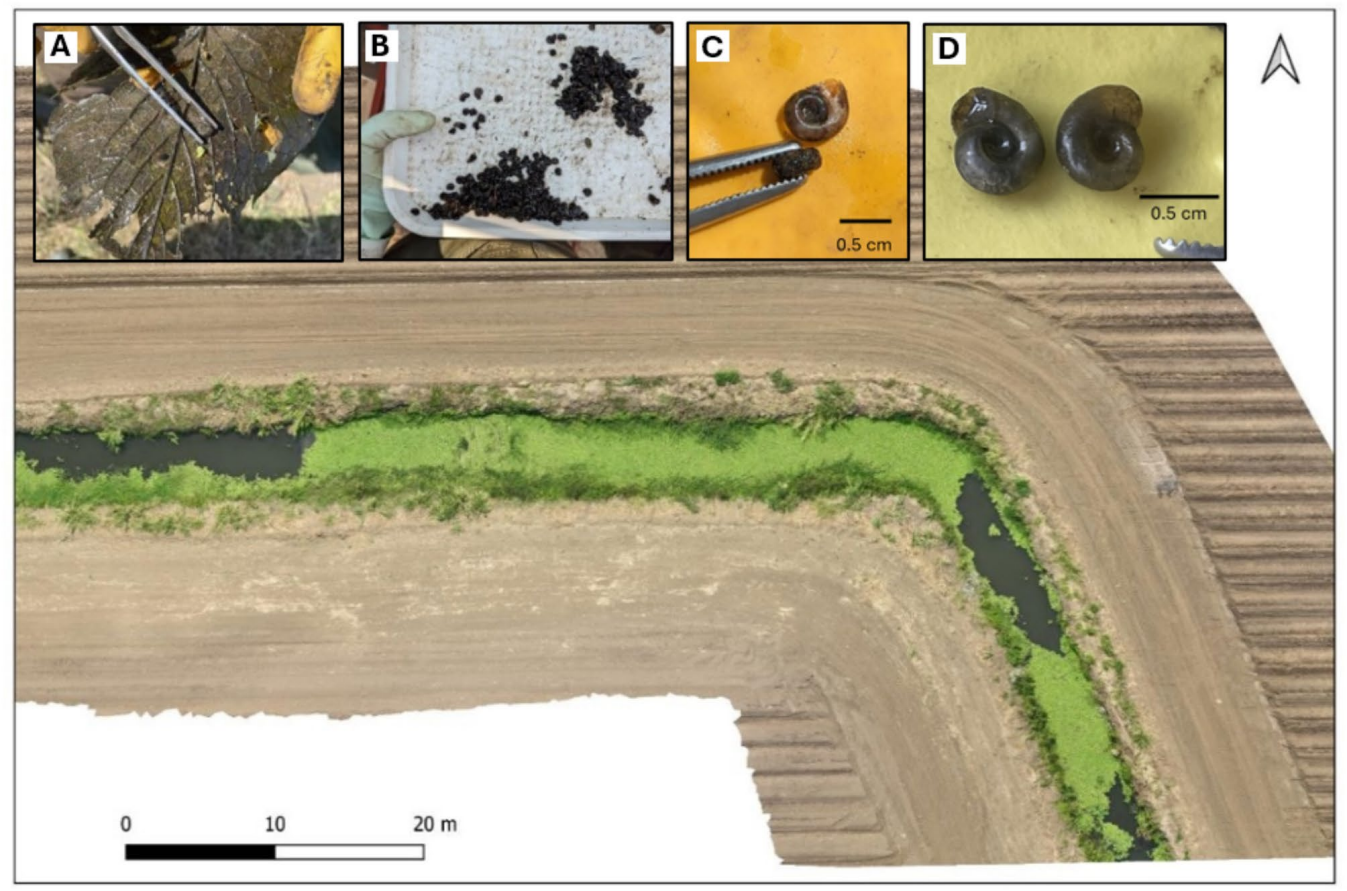
A drone imagery of a semi-aquatic snail habitat (S16.17069, E34.8487) in Chikwawa which yielded a total of 122 *Biomphalaria* snails as presented with QGIS version 3.22.1 mapping software. The habitat is a narrow earth-lined irrigation canal/drain with deep, slow flowing water (mean pH = 8.2, mean temperature = 29.4 °C, mean conductivity = 1001 µS/cm, mean TDS = 501 ppm). Here, the snails were found floating on the surface or attached to the underside of water lilies (**A**) being collected in large numbers (**B**) with their shells shown in apical (**C**) and, apertural and umbilical (**D**) views.

**Fig. 3:**
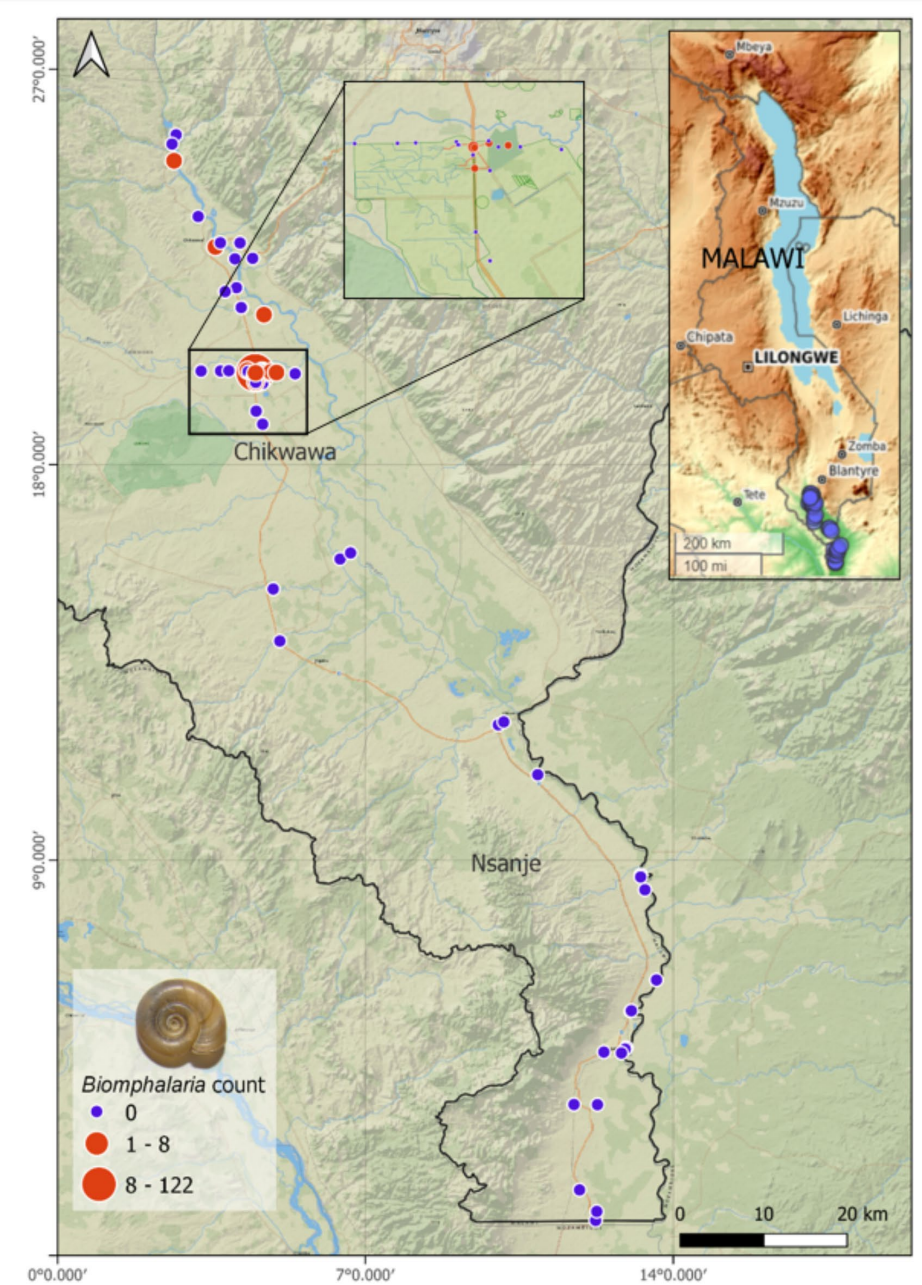
Observed locality, distribution and abundance of *Biomphalaria* within the study area as presented with QGIS version 3.22.1 mapping software.

From the malacological surveys, several extant *Biomphalaria* populations were found to occur in Chikwawa, observed for the first time (Fig. 2) and a seminal report of this genus in the Lower Shire Valley. Of the 45 sites sampled, 11 sites (23%) were found to contain these snails. A total of 144 *Biomphalaria* were collected from irrigation canals in Chikwawa (Supplementary Table 1). The highest abundance of *Biomphalaria* (*n =* 122) was found in site number 17 representing almost 85% of the observed snail population from the sampled irrigation canals. A total of 22 *Biomphalaria* were recorded in low abundances in other sites (*n* ≤ 8 in 10/28 sites). In Nsanje, there were no *Biomphalaria* recorded during the survey (Fig. 3). No collected *Biomphalaria* were found to be shedding *Schistosoma* spp. cercariae during cercarial shedding analysis.

### Molecular characterization and molecular xenomonitoring of collected *Biomphalaria*

Genomic DNA (gDNA) was successfully isolated from all eight *Biomphalaria* specimens, all of which were identified as *B. pfeifferi* through *cox*1 analysis^29^. All eight *B. pfeifferi cox*1 sequences were submitted to the GenBank repository (accession numbers OR880274 - OR880281). No evidence of *B. pfeifferi* infection with *S. mansoni* (or any other species of Trematoda) was found using molecular xenomonitoring.

### How is the occurrence of *B. pfeifferi* snails associated with variation in elevation and aquatic physicochemical conditions in lower Shire?

1. Variable association

**Fig. 4:**
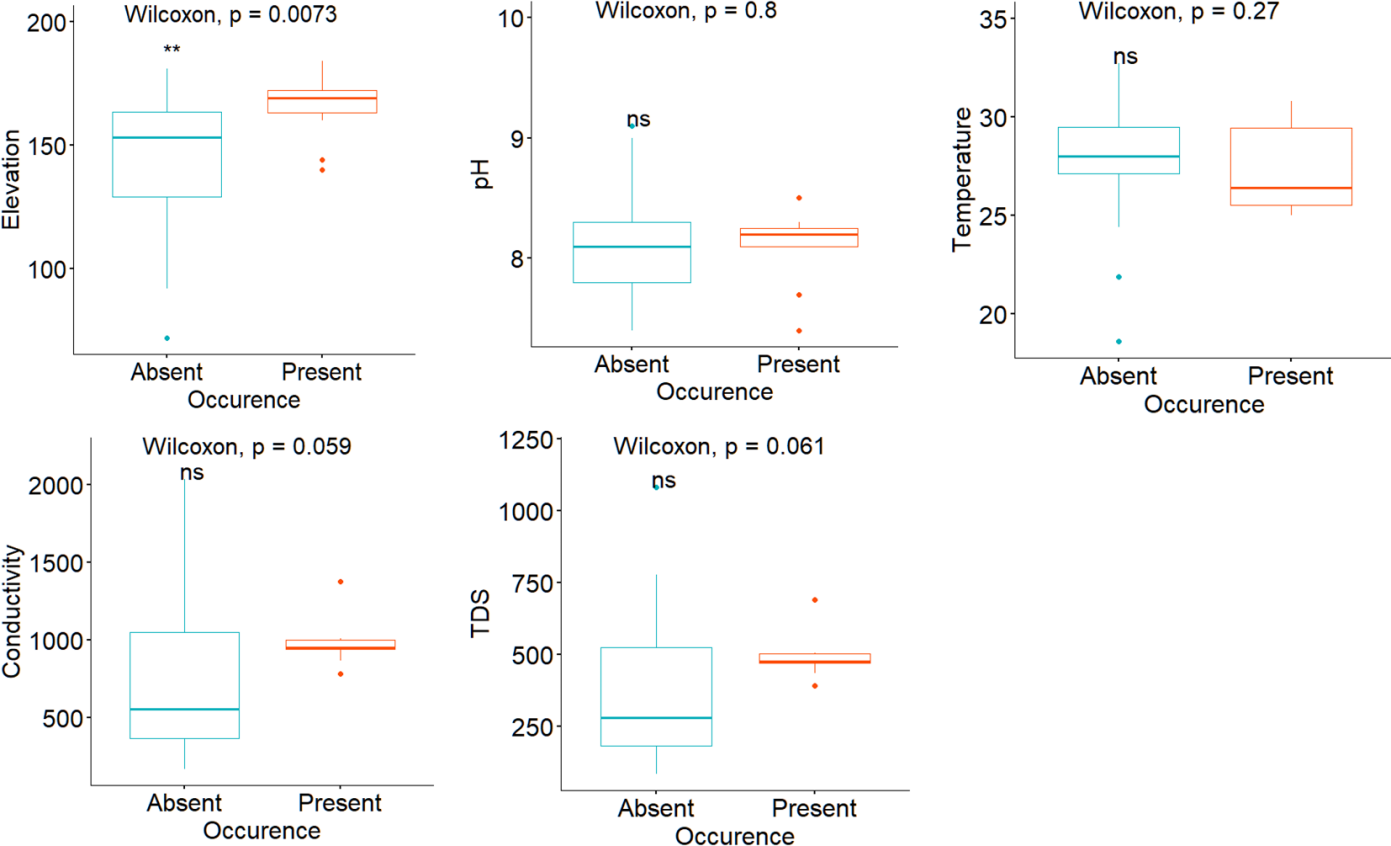
Box plot of the explanatory variables and *B. pfeifferi* occurrence in the study area.

Upon the molecular identification and confirmation of the *B. pfeifferi*, Fig. 4 below indicates the ranges associated with snail’s presence or absence across the surveyed area. From the plot, generally *B. pfeifferi* presence is probable if elevation is around > 150 m, pH is < 8.5, temperature is between > 25 to < 31 °C. In addition, conductivity of around 1000 µS/cm and TDS of around 500 ppm are associated with *B. pfeifferi* presence.

2. Variable importance

**Fig. 5:**
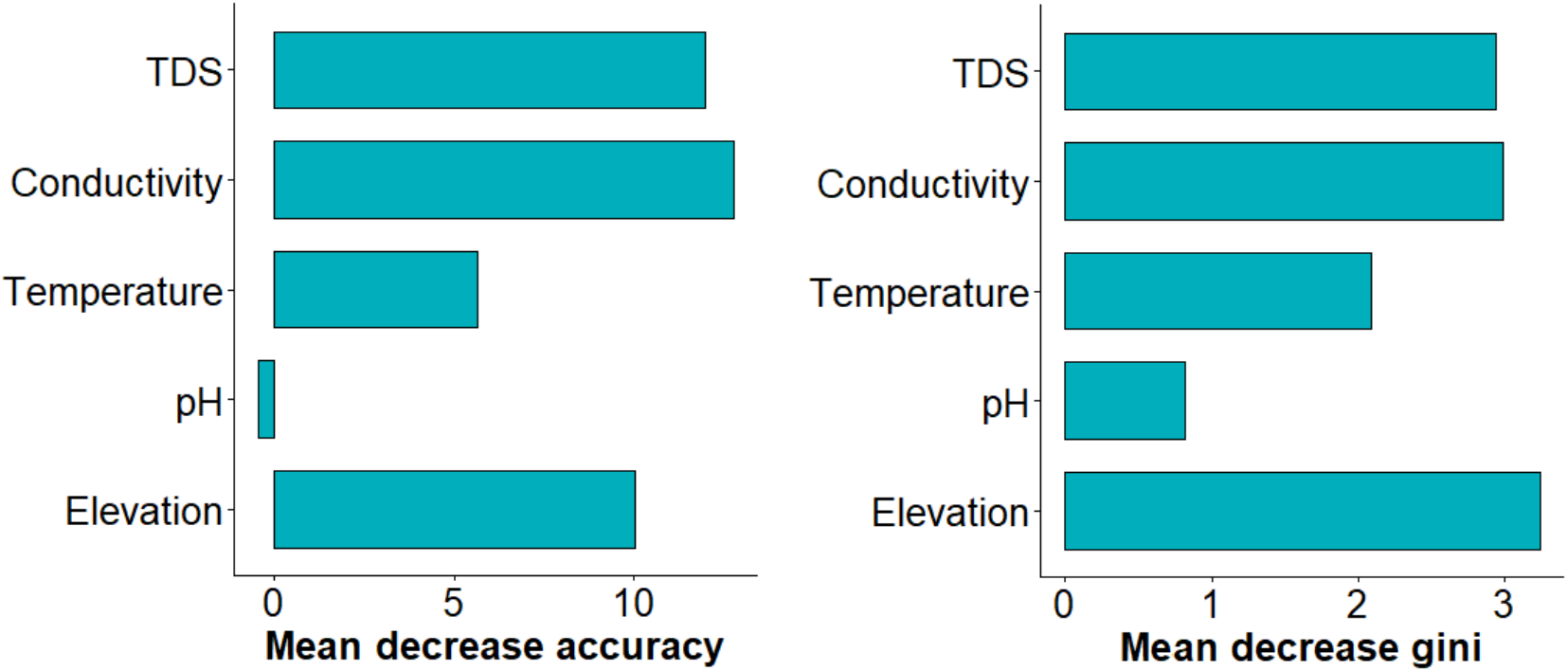
Variable importance plot ranking the explanatory variables (y-axis) based on their predictive power and contribution to the accuracy of the predicted *B. pfeifferi* presence and absence in the study area by the RF model. A higher MDG value indicates that the variable was significant in distinguishing between classes in the model. A variable with a higher MDA contributed more to the predictive accuracy of the model, as its absence (or randomization) lead to a significant drop in model performance.

**Fig. 6:**
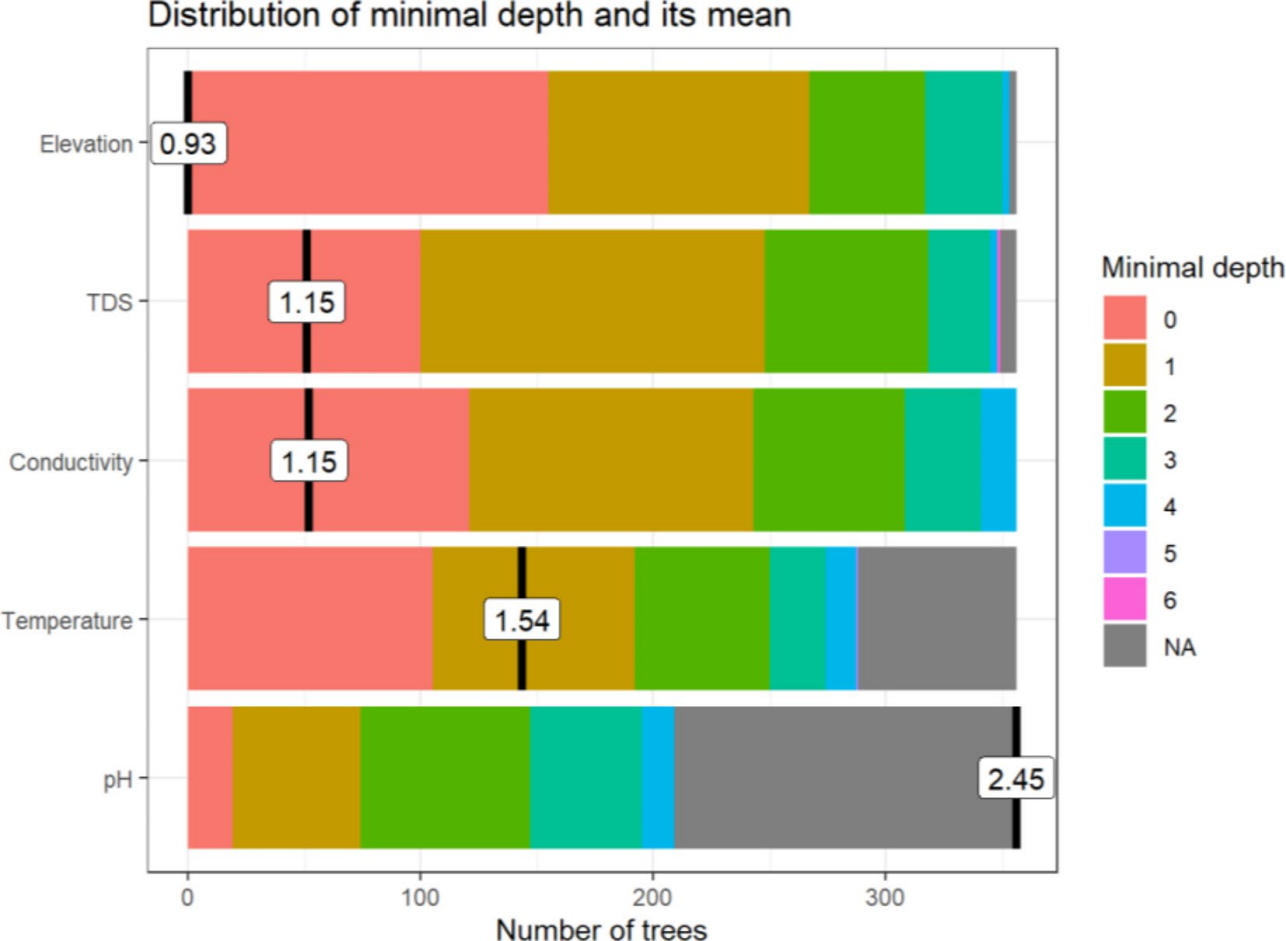
The minimal depth plot indicates the impact of the explanatory variables on the predictive ability of the RF model. Lower values indicate the most important variables in determining the decision boundary relative to the number of trees. Elevation, TDS, conductivity, temperature and pH contributed to the decision trees when 150, 100, 110, 100, and 10 trees were split in the ensemble, respectively. Noticeably, from >200 trees onwards, pH and temperature were not selected for splitting in the classification trees. Note that the mean of the distribution is marked by a vertical bar with a value label on it (the scale for it is different than for the rest of the plot), and the scale of the X axis goes from zero to the maximum number of trees in which any variable was used for splitting.

Figure 5 provides the most and least important variables in explaining the spatial presence and absence of *B. pfeifferi* in the study area. From the plot, conductivity contributed significantly to the prediction accuracy of the RF model, followed by TDS, then elevation, temperature and lastly, pH. Based on the MDA, conductivity (MDA = 12.78), TDS (MDA = 11.98) and elevation (MDA = 10.03) rank highly. From the fitted model, the prediction accuracy decreased significantly when the TDS and conductivity variables are removed from the model. With an MDA of 5.65, temperature ranked moderately, whereas pH ranked lowest, with an MDA of -0.45. Figure 6 shows that elevation, conductivity and TDS had a higher MDG of 3.24, 2.98 and 2.93, respectively. This indicates that these variables were more important for the model’s predictive power. While temperature had a moderate MDG of 2.08, in contrast, pH had low MDG of 0.81, indicating less impact of the former on the accuracy of the *B. pfeifferi* prediction model.

**Fig. 7:**
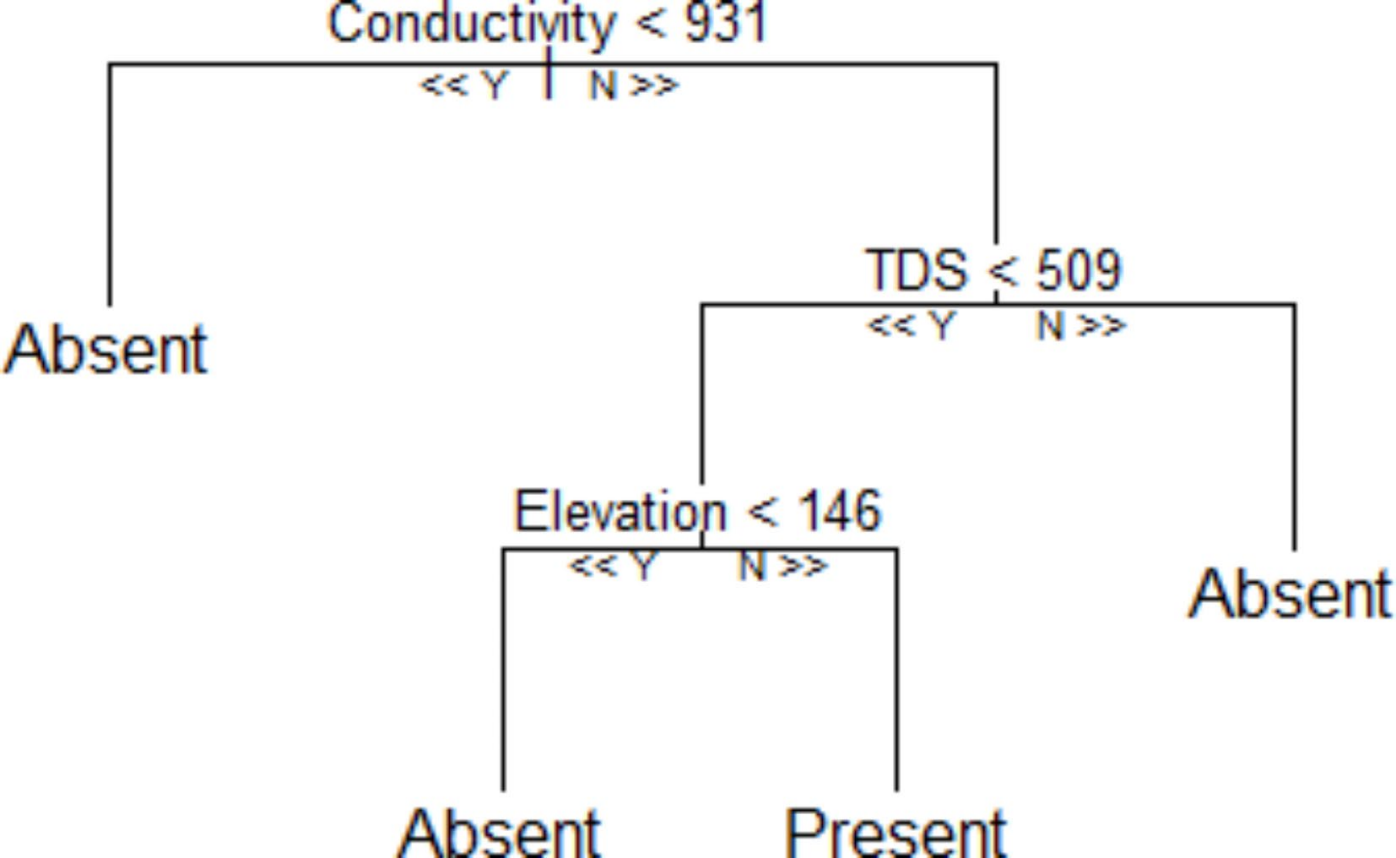
The fitted RF classification tree model predicts the presence or absence of *B. pfeifferi* in lower Shire.

Figure 6 below shows the relative importance of the explanatory variables in predicting *B. pfeifferi* based on the depth at which the variables appeared in any of the decision trees when constructing the forest. From Fig. 7, it can be observed that elevation, TDS, conductivity and temperature had relatively low minimal depth. This observation differs from that of pH.

3. RF model prediction

The RF classification tree for *B. pfeifferi* presence-absence in the Lower Shire is set out in Fig. 7. From the tree, conductivity is the root, indicating that it was measured as the most important variable for the prediction of *B. pfeifferi* occurrence. From the RF prediction, *B. pfeifferi* presence is probable if conductivity > 931 µS/cm, TDS < 509 ppm and elevation > 146 m. This is consistent with the in situ aquatic physicochemical and topographic conditions observed at the sites where *B. pfeifferi* occurred (Supplementary Table 1). Collectively, conductivity, TDS and elevation were important for the snail’s presence or absence in the study area.

### Model performance

RF model performance was evaluated using accuracy, precision, recall (sensitivity) and F1 metrics calculated from the confusion matrix (Table 3). The accuracy of the model on testing data was 0.8 (80%) with 95% CI (0.4439, 0.9748). The precision, recall and F1 scores on the ‘positive’ class (absent) were 0.7778 (~ 78%), 1 (100%) and 0.8750 (~ 88%), respectively. Together, these high values indicate that the model performed well for the given presence/absence prediction task (Supplementary Fig. 3 and Fig. 4, Supplementary Table 4). Consider, for example, the F1 score. A high F1 score suggests that the model balanced precision and recall, and vice-versa^41^.

**Table 3:**
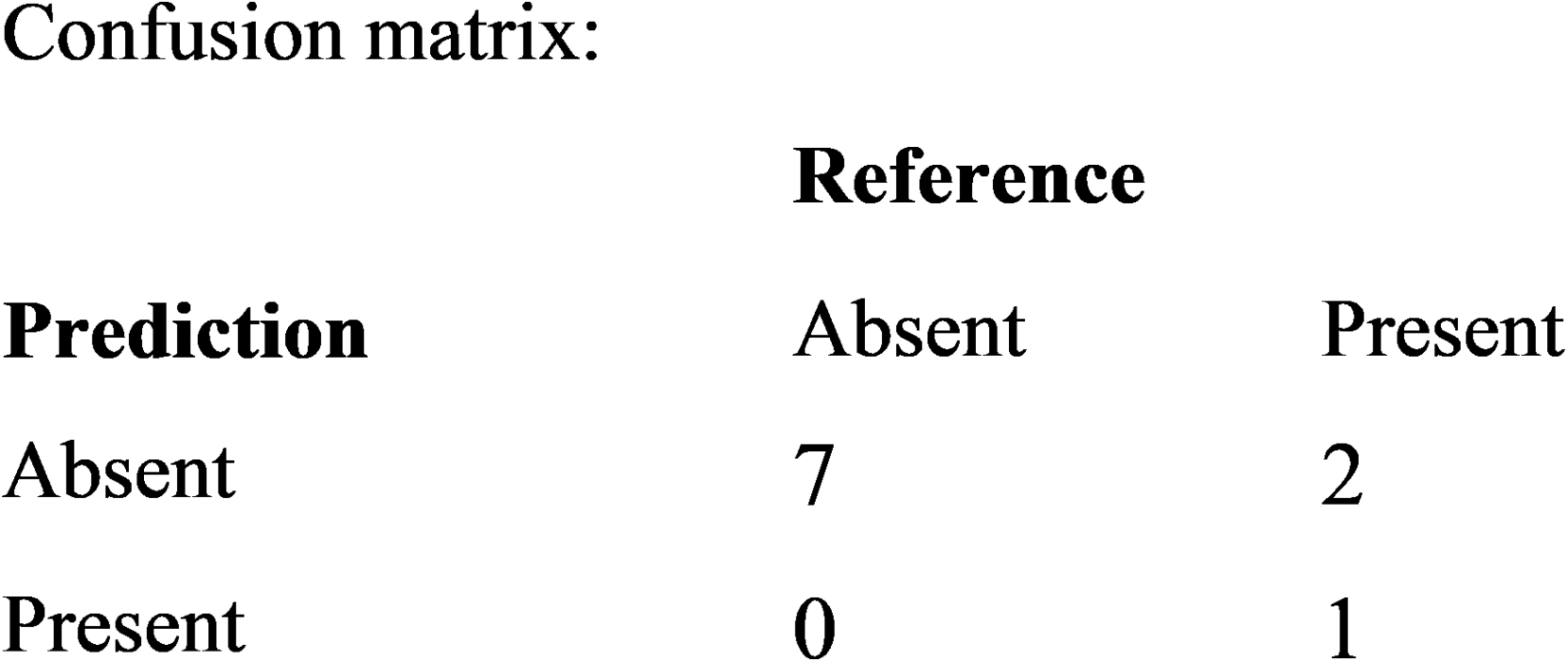
Confusion matrix and statistics for the model on test data.

## Discussion

Using a combination of malacology surveys, geospatial techniques and molecular analyses, our study has detected and identified several *B. pfeifferi* populations for the first time in Chikwawa District. To our knowledge, this constitutes the first formal report of this species in the Lower Shire Valley, an important finding given its well-known role in intestinal schistosomiasis transmission elsewhere (Supplementary Fig. 1, Fig. 2 and Fig. 5). We posit that across the Lower Shire Valley, this species is difficult to find because the population size and density are low, and the Shire River landscape is complex with many ephemeral water habitats that may be colonized by snails outside of the dry season. The RF model results indicate that *B. pfeifferi* occurrence varies across fine-scale water chemistry and physical environmental gradients and is related with conductivity, TDS and elevation (Supplementary Fig. 3 and Fig. 4). Conversely, the results show that while temperature and pH are related with the spatial presence and absence of *B. pfeifferi*, the association of the snails to these variables, however, is weak. This suggests that across the study area, water pH and temperature moderately influence *B. pfeifferi* occurrence. Because the predictive power of elevation and water conductivity and TDS was high, the RF model suggests that these three variables are the most important predictors of *B. pfeifferi* spatial presence/absence in lower Shire. This finding supports evidence from previous observations^5,23,42^.

The study found that, generally, elevations from 160 to 180 m, aquatic TDS between 400 and 600 ppm (mg/L) and aquatic conductivity ranging from 800 to 1000 µS/cm are positively associated with *B. pfeifferi* presence across the lower Shire (see Figs. 4, 5, 6 and 7). Of note, water conductivity and TDS are correlated^41^, and conductivity is an indicator of salinity^43^. Collectively, it seems possible that *B. pfeifferi* is sensitive to aquatic salinity and can only tolerate a specific salinity range. This implies that salinity change, for example, due to human disturbances such as farming^44,45^, evaporation, run-off and flooding can affect the presence of the *B. pfeifferi* across the study area. Considering that the water physicochemical conditions across the study area exhibited spatial variation along an elevation gradient, it naturally follows that the distribution of *B. pfeifferi* is disjunct and non-uniform. Thus, this study: (1) revealed that *B. pfeifferi* is present in Chikwawa, (2) showed that *B. pfeifferi* was abundant at sites with slightly alkaline water, (3) asserts reports of *B. pfeifferi* snails having a narrow tolerance range for water conductivity and alkalinity^26^, and (4) supports the idea that hydrogen ion concentration (pH) is a weak limiting factor for the species distribution^22^. Overall, the observed *B. pfeifferi* occurrence and abundance appears to be influenced by the compound interactions among these aquatic physicochemical and environmental factors. For example, it is well known that the water temperature affects conductivity readings^46,47^ and TDS and conductivity are correlated^42,47,48^. In the variable importance plot, the close ranking of TDS and conductivity is hardly surprising because they both essentially measure salinity levels^49^. Taken together, it emerges that saltiness of water provides the best prediction of the snail’s occurrence.

The confusion matrix indicates that the accurate prediction of *B. pfeifferi* presence by the RF model proved difficult. The RF model performed extremely well in predicting the absence of the *B. pfeifferi*. Since misclassification errors were reasonably high for presence outcome, the model was at its worst when predicting presence (Supplementary Table 2). A possible explanation for this might be that the snails have low occurrence in “suitable habitat”. Very few presences were recorded in suitable habitat (Supplementary Table 1). Another reason may be the lack of adequate data of presence and absence records. There would therefore seem to be a definite need to explore methods of improving spatially-naive RF models for predicting *B. pfeifferi*, and for more records of *B. pfeifferi* presence/absence across lower Shire. Overall, the RF results are acceptable, given that 80% of the influence of the aquatic chemistry and elevation on *B. pfeifferi* occurrence across lower Shire can be explained by the fitted RF model. One notable strength of the RF algorithm is its ability to discover and explore non-linear relationships in complex, environmental data with high accuracy, even with limited sample size^31–33^. Another strength is its ability to determine variable importance and find an optimal solution while averting over-fitting and collinearity between variables, issues generally associated with traditional parametric models^31,32^. In important papers on RFs, Breiman^31,37^ provides empirical evidence for this assertion.

Finally, to augment our geospatial findings, a wider phylogenetic analysis of our *cox*1 sequences was recently reported by Archer et al.^29^, revealing strong affiliations with Mangochi District (Malawi) and Zimbabwe populations. It is reasonable to assume a relatively recent downstream dispersal of *B. pfeifferi* from Mangochi to Chikwawa, notably haplotype 2 as originally identified by Al-Harbi et al.^9^. Whilst Archer et al.^29^ detected a single *B. pfeifferi* shedding cercariae of *S. mansoni* in their shoreline surveys, interpretation of our molecular xenomonitoring findings presented here is more equivocal. The most recent epidemiological survey for intestinal schistosomiasis, conducted in September 2023 by Chiepa et al.^50^, is noteworthy. Using urine-circulating cathodic antigen dipsticks, the study sampled 1,134 school-aged children from 21 government-owned primary schools, identifying the highest local prevalence of 18.5% at Tomali Primary School. Of note, Tomali Primary School is located closest to sampling site 17, less than 5 km away, but to unequivocally prove autochthonous transmission, the search for locally shedding snails continues across the Chikwawa floodplain continues.

## Conclusions

Our study presents the first report and formal record, with geovisualisation and spatial modelling, of *B. pfeifferi* within Chikwawa District. Better predicting and noting the presence and abundance of *B. pfeifferi* in future is an important step towards developing locally appropriate interventions to reduce intestinal schistosomiasis transmission.

## Electronic supplementary material

Below is the link to the electronic supplementary material.

Supplementary Material 1

## Data Availability

The genetic sequences generated and/or analysed during the current study are available in the GenBank repository, [https://www.ncbi.nlm.nih.gov/genbank/ and Accession numbers OR880274 - OR880281]. The survey dataset that supports the findings of this study are available in the supplementary materials.
