# Supplementary material for: A first report of Biomphalaria pfeifferi in the Lower Shire Valley, Southern Malawi, a major intermediate snail host species for intestinal schistosomiasis
Source: Sci Rep. 2025 Feb 15;15:5603. doi: 10.1038/s41598-025-88930-4 (PMC11830094; doi:10.1038/s41598-025-88930-4)

A first report of *Biomphalaria pfeifferi* a major intermediate snail host species for intestinal schistosomiasis in the Lower Shire, Southern Malawi

Clinton Nkolokosa, Rex Mbewe, James Chirombo, Michelle C. Stanton, Christopher M. Jones, Peter Makaula, Gladys Namacha, Blessings Chiepa, Patrick Ken Kalonde, Chifuniro Baluwa, Kennedy Zembere, Aisha Kambewa, Chikumbusko Chiziwa Kaonga, John Archer, Alexandra Juhász, Lucas J. Cunningham, Julie-Anne Akiko Tangena and J. Russell Stothard

Supplementary Table 1

ID	Location	District	Elevation (m)	pH	Temperature (°C)	Conductivity (µS/cm)	TDS (ppm)	*B. pfeifferi* Present?	
1	Ndindi Marsh Marka	Nsanje	92	7.4	27.4	433	212	0	No
2	Wetland Marka	Nsanje	106	8.8	18.6	362	184	0	No
3	Nsanje Marka	Nsanje	146	7.9	29.0	168	83	0	No
4	Nyamkotola River	Nsanje	155	8.8	31.0	556	279	0	No
5	Nsanje Port	Nsanje	117	7.6	29.0	337	119	0	No
6	Malindi River	Nsanje	162	9.1	29.2	444	222	0	No
7	Nyamikoto wetland	Nsanje	129	8.3	32.3	853	427	0	No
8	Nyamadzete bridge	Nsanje	143	9.0	29.7	553	279	0	No
9	Nsanje Port rice paddy	Nsanje	132	7.8	30.9	1006	502	0	No
10	Nsanje Chididi HUGS 04	Nsanje	95	8.0	27.9	446	223	0	No
11	Nsanje HUGS 03	Nsanje	98	8.3	30.6	331	166	0	No
12	Mtayamoyo Bangula	Nsanje	126	7.7	27.4	314	157	0	No
13	Doko Mtayamoyo close to Shire River	Nsanje	130	7.7	27.8	380	192	0	No

Supplementary Table 1

ID	Location	District	Elevation (m)	pH	Temperature (°C)	Conductivity (μS/cm)	TDS (ppm)	*B. pfeifferi*	Present?
14	Phokera	Nsanje	134	7.9	27.8	311	155	0	No
15	Nyakamba River	Nsanje	93	8.2	31.6	1062	532	0	No
16	River, new road and bridge diversion	Nsanje	72	8.2	29.5	1280	640	0	No
17	Water canal	Chikwawa	170	8.2	29.4	1001	501	122	Yes
18	River HUGS site near Bereu	Chikwawa	173	7.9	27.7	463	233	0	No
19	Kamuzu bridge close to Shire River	Chikwawa	179	8.5	30.8	950	474	1	Yes
20	Large body of water before Thabwa	Chikwawa	180	8.5	27.0	267	132	0	No
21	Water canal	Chikwawa	140	8.1	25.4	1378	689	1	Yes
22	Water canal	Chikwawa	140	8.3	28.1	1254	620	0	No
23	Water canal	Chikwawa	140	7.8	27.1	1254	638	0	No
24	Water canal	Chikwawa	144	8.1	28.4	866	434	1	Yes
25	Water canal	Chikwawa	154	7.8	21.9	1413	710	0	No
26	Water canal	Chikwawa	162	8.1	29.4	1574	776	0	No

Supplementary Table 1

ID	Location	District	Elevation (m)	pH	Temperature (°C)	Conductivity (µS/cm)	TDS (ppm)	***B. pfeifferi***	**Present?**
27	Water canal	Chikwawa	167	8.1	24.4	1332	665	0	No
28	Water pumping and storage	Chikwawa	164	8.3	28.6	356	178	0	No
29	Small pond close to road	Chikwawa	167	7.9	25.6	976	489	0	No
30	Water canal across Nyala Park entrance	Chikwawa	166	8.2	28.2	376	180	0	No
31	Water canal entering Nyala Park	Chikwawa	160	8.1	29.6	1004	502	3	Yes
32	Water canal inside Nyala Park	Chikwawa	184	8.5	25.0	941	469	1	Yes
33	Crocodile Dam Nyala Park	Chikwawa	181	8.4	27.1	1159	580	0	No
34	Pond inside Nyala Park	Chikwawa	174	7.4	25.4	784	392	1	Yes
35	Water canal inside Nyala Park	Chikwawa	166	8.2	25.6	937	469	8	Yes
36	Water canal inside Nyala Park	Chikwawa	166	8.2	26.4	949	473	1	Yes
37	Water canal outside Nyala Park	Chikwawa	170	7.7	26.2	1008	504	3	Yes
38	Water canal	Chikwawa	177	7.7	27.1	408	207	0	No
39	Water canal	Chikwawa	169	8.3	29.5	961	480	2	Yes

Supplementary Table 1

ID	Location	District	Elevation (m)	pH	Temperature (°C)	Conductivity (µS/cm)	TDS (ppm)	***B. pfeifferi***	Present?
41	Water canal near Mwanza River	Chikwawa	167	8.2	26.8	913	457	0	No
42	Static water under small bridge	Chikwawa	152	7.8	27.4	927	464	0	No
43	Large body of water near Shire at Chikwawa	Chikwawa	159	8.1	30.2	796	452	0	No
44	Kasinthula	Chikwawa	156	7.5	29.3	762	326	0	No
45	Kasinthula sugar irrigation Site A	Chikwawa	162	8.5	27.1	332	165	0	No
46	Kasinthula sugar irrigation Site B	Chikwawa	162	8.3	32.7	2035	1081	0	No

Supplementary Figure 1

Occurrence of *B. pfeifferi* across a gradient of *in situ* physicochemical properties of water and topographical variables recorded at all sampling sites (n = 45).

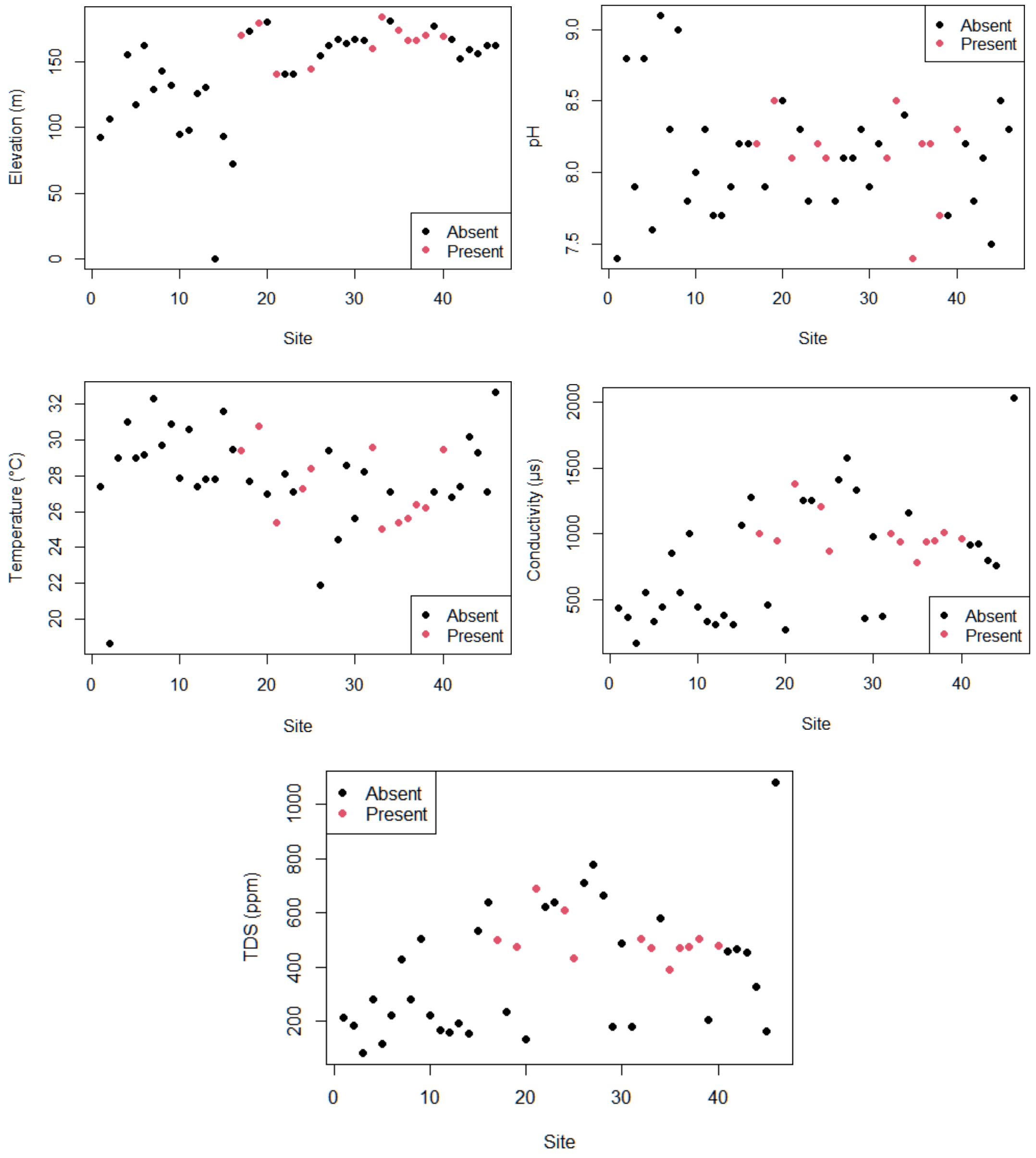

Supplementary Figure 2

In situ physicochemical properties of water and topographical variables along north (Chikwawa) to south (Nsanje).

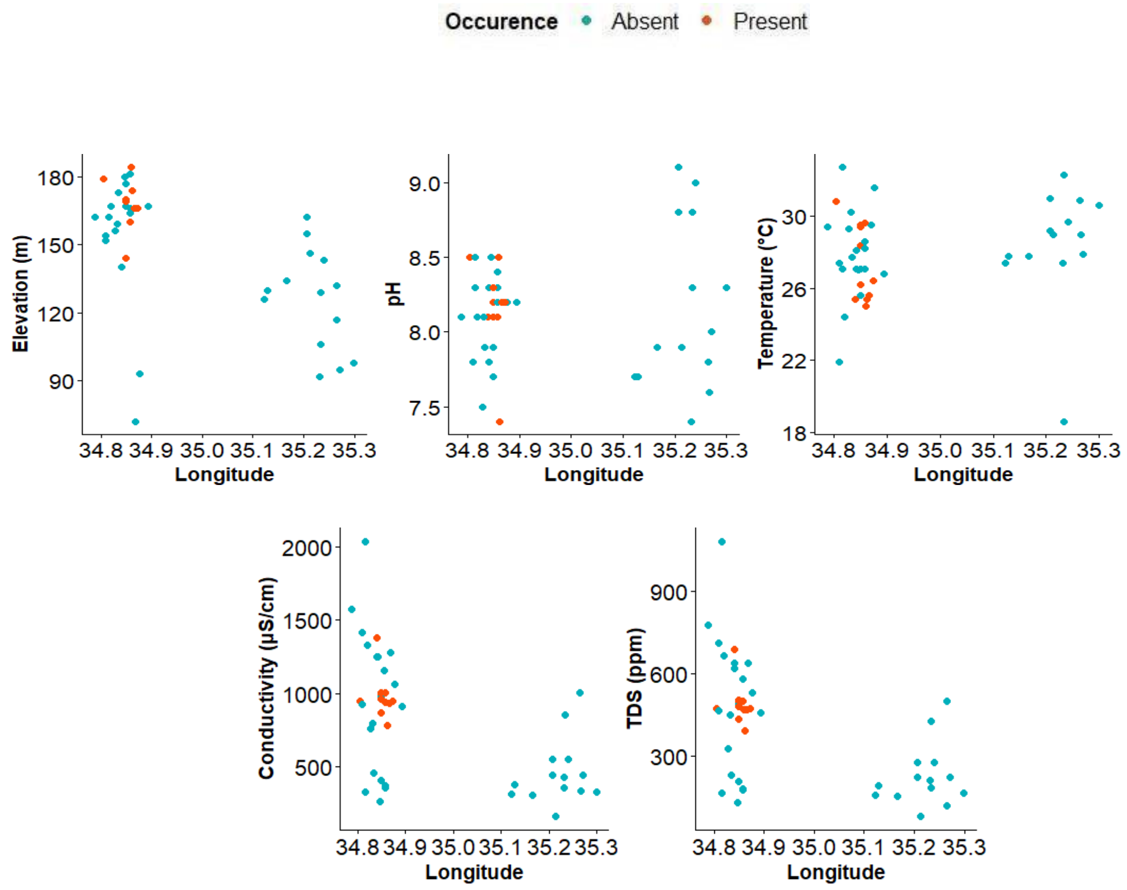

Supplementary Figure 3

Error vs number of trees shows a plot of the out-of-bag (OOB) error as the number of trees in the RF model increased. The OOB error is an estimate of prediction error on test data. Of interest here is the sharp decline of the line as the number of trees increases towards 100, clearly indicating that the error rate decreased when more trees were added. The error rate generally levelled off after 200 trees and reached lowest, indicating that this was the optimum number of trees and that beyond this point the addition of more trees would have no significant improvement on the model's performance.

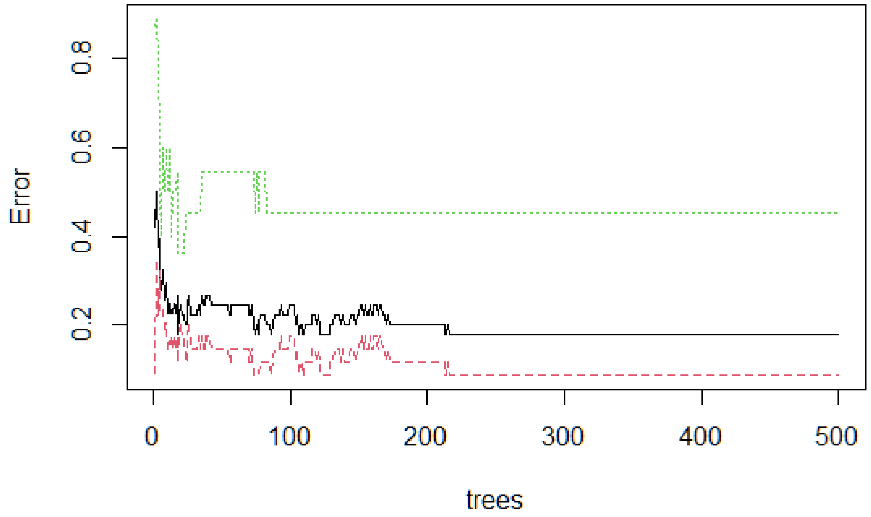

Supplementary Table 2

A confusion matrix showing how many of the RF classifiers predictions were correct and where the model was confused (i.e., made incorrect predictions). From the table, three sites randomly sampled by the model were wrongly predicted to have no *B. pfeifferi* and 1 randomly sampled site was wrongly predicted to host the snails. Clearly, the RF model struggled to predict the presence of *B. pfeifferi* (class error = 0.375 or ~37.5%). There is ten-fold error in the presence predictions. However, this must be regarded with caution since there is a significant class imbalance between the absence and presence classes. In contrast, with a very low class error of 0.037 or 3.7%, the model showed greater accuracy (96.3%) in predicting *B. pfeifferi* absence. Overall, prediction of presence yielded highest misclassification, suggesting that accurate prediction of *B. pfeifferi* presence proved difficult.

OOB estimate of error rate: 11.43%

Confusion matrix:

	Absent	Present	Class error
Absent	26	1	0.037
Present	3	5	0.375

Supplementary Figure 4

Partial dependence shows the values for each variable that have a positive influence for *B. pfeifferi* presence. In other words, possible optimum ranges of conductivity, elevation, pH, temperature and TDS for *B. pfeifferi* presence. From the charts, the marginal effect of each variable on the predicted outcome of *B. pfeifferi* presence/absence while holding all other variables constant is presented. Note on y-axis, a negative value means for that particular value of the explanatory variable it is less likely to predict presence on that observation. On the other hand, a positive y-axis value means higher probability of predicting presence.

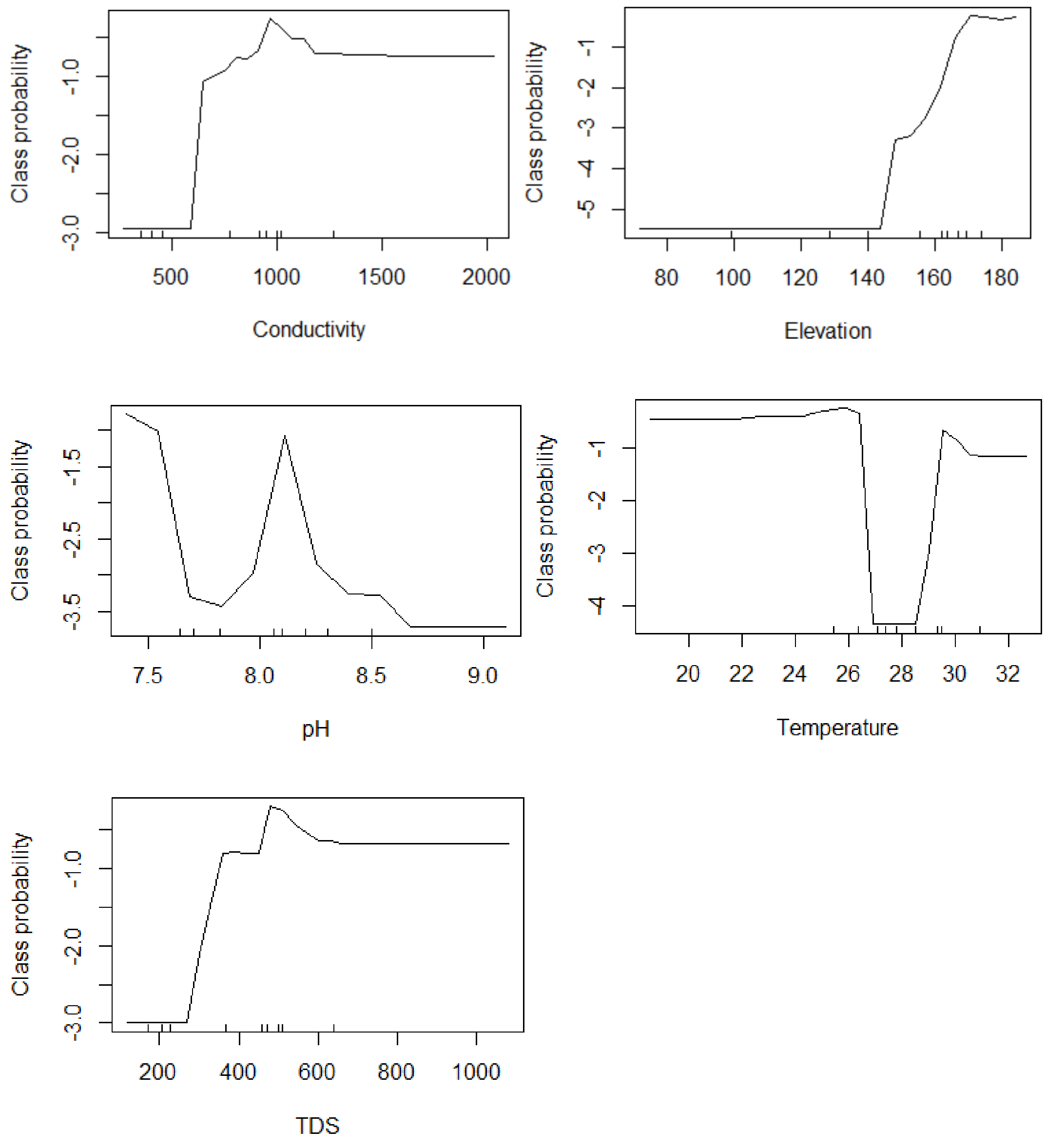

Supplementary Table 3

The mean minimal depth indicates the depth at which the explanatory variable appears in any of the decision trees in the RF prediction.

Variable	Mean minimum depth	No. of nodes	No. of trees	Times a root	p_value
Conductivity	1.1746	571	378	113	0.0000
Elevation	0.9343	546	353	121	0.0001
pH	2.4469	265	209	19	1.0000
TDS	1.1500	533	349	100	0.0011
Conductivity	1.1545	567	356	121	0.0000
Temperature	1.5434	451	288	105	0.8703

Supplementary Figure 5

Distribution and abundance of other snails recorded during the malacological survey in Chikwawa and Nsanje.

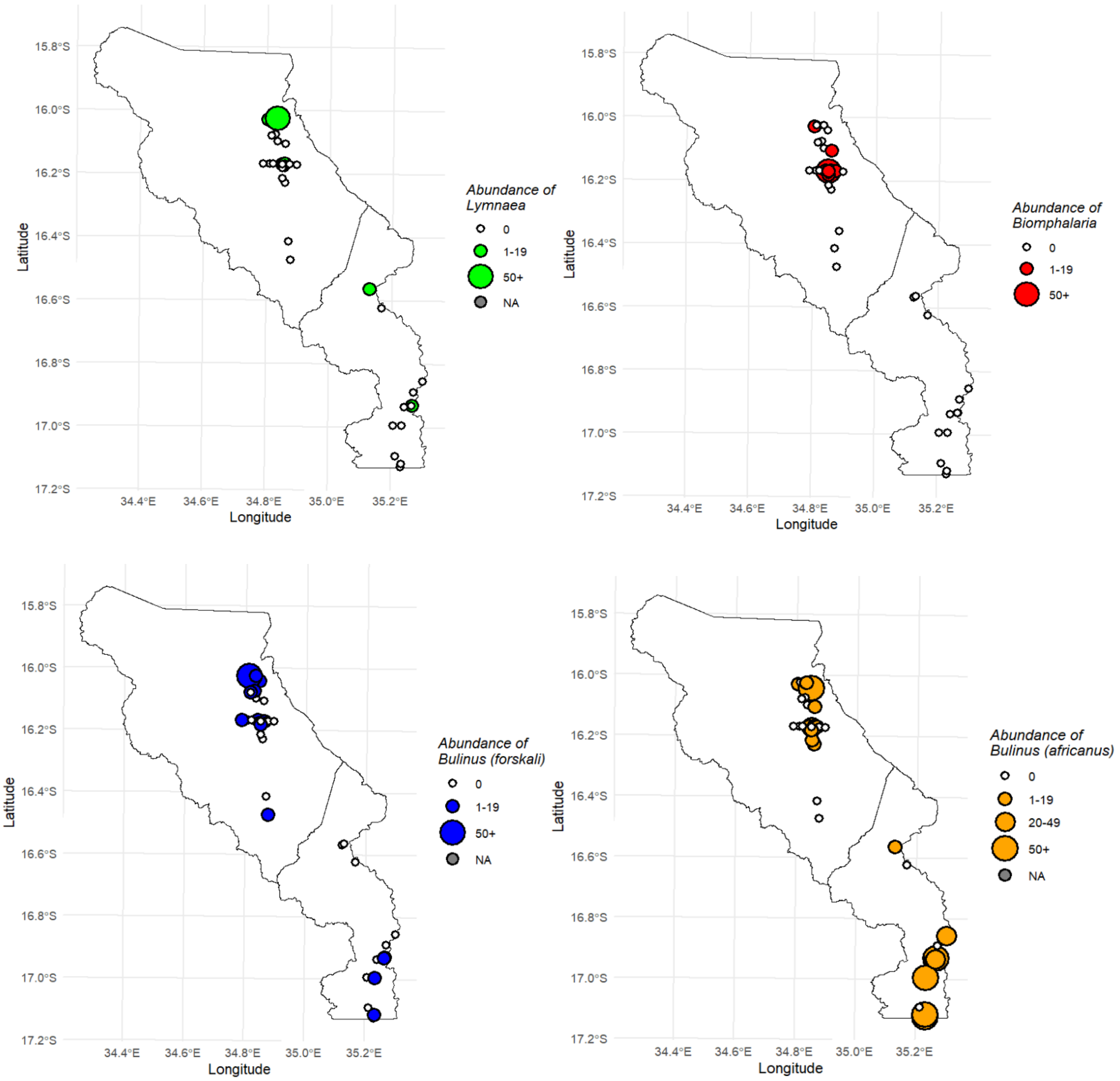

Presence records of snails *Melanoides* and *Lanistes*

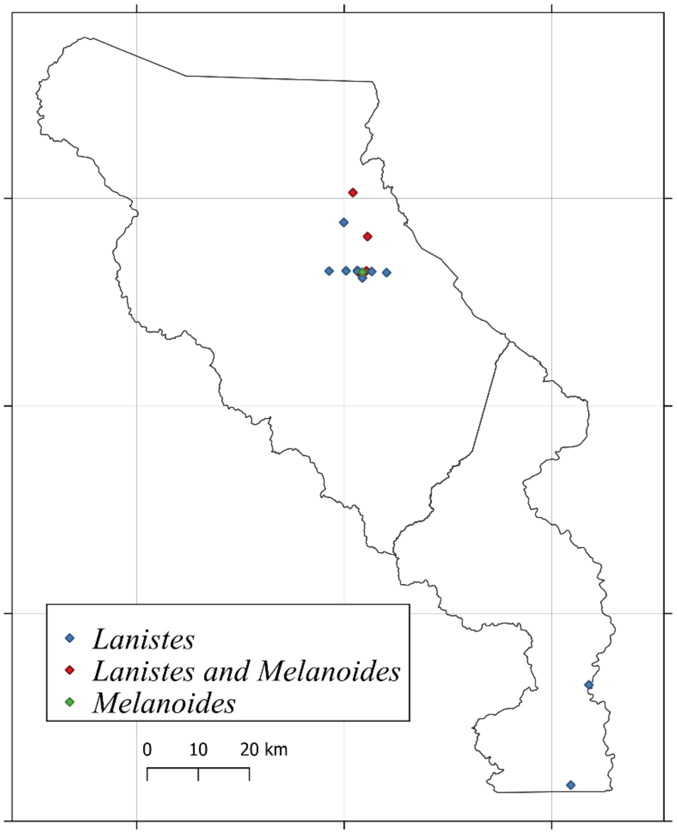

Supplement: Supplementary file 1 — Supplementary Material 1 [file 41598_2025_88930_MOESM1_ESM.pdf]
